# Evaluation of the Appropriate Use of Coronary Computed Tomography Angiography: A Retrospective, Single-Center Analysis

**DOI:** 10.3390/jcdd9060180

**Published:** 2022-06-04

**Authors:** Katharina Birkl, Christoph Beyer, Fabian Plank, Gudrun Maria Feuchtner, Guy Friedrich

**Affiliations:** 1 Department of Radiology, Innsbruck Medical University, 6020 Innsbruck, Austria; katharina.birkl@i-med.ac.at (K.B.); christoph.beyer@i-med.ac.at (C.B.); gudrun.feuchtner@i-med.ac.at (G.M.F.); 2 Department of Internal Medicine III–Cardiology and Angiology, Innsbruck Medical University, 6020 Innsbruck, Austria; fabian.plank@tirol-kliniken.at

**Keywords:** coronary computed tomography angiography, appropriate use, coronary artery disease, invasive coronary angiography, percutaneous coronary intervention

## Abstract

Purpose: We assessed the application of appropriate use criteria of coronary computed tomography angiography (CCTA) in comparison to invasive coronary angiography results and revascularization rates in patients with coronary artery disease (CAD). Methods: 1305 patients referred to invasive coronary angiography (ICA) after CCTA were evaluated retrospectively. The primary indication for CCTA was assessed according to the consensus for intermediate-risk (15–85% pre-test probability) into appropriate (A), inappropriate (I), and uncertain while referring to published guidelines. Patients’ risk factors, angina, and heart failure symptoms (Canadian Cardiovascular Society classification (CCSC), New York Heart Association (NYHA); clinical data; and ICA results were gathered. Results: Of 1305 patients referred to CCTA prior to ICA, 496 (38.0%) were appropriate, 766 (56.9%) inappropriate, and 43 (3.3%) uncertain. Of 766 patients with inappropriate CCTA referrals, 370 (48.3%) were classified as “inappropriately low” (<15% pre-test probability) and 396 (51.7%) as “inappropriately high” (>85%) in regard to the recommended CCTA utilization. Sub-analysis of the adherence to the appropriate use criteria did not differ between the source of the referring physicians (intramural tertiary, private practice primary care, or external secondary care hospitals). Obstructive CAD with subsequent revascularization rates (total of 39.2%) did not differ significantly between the appropriate (38.3%), inappropriate (41.0%), or uncertain (23.3%) groups (*p* = 0.068). Conclusion: The total coronary revascularization rate after CCTA was 39.2% and not different among low, intermediate, and pre-test probability groups. These findings support the role of CCTA as an excellent gatekeeper in patients with suspected obstructive CAD even beyond pre-test probability calculation models.

## 1. Introduction:

The primary and accurate detection of coronary artery disease has always been a challenging task for clinicians but remains paramount in the practice of cardiology [1]. Cardiovascular disease is still the leading global cause of death, with more than 17 million people annually, thereof over 4 million in Europe [2]. 

Apart from primary and secondary prevention of CAD, an early and correct diagnosis is crucial to reducing mortality and healthcare costs. Pre-test probability models for CAD were published earlier and, due to over-and underestimated risk rates, revised and republished [3,4,5,6,7]. 

Coronary computed tomography angiography has emerged over the past decade as a non-invasive tool for the diagnosis of coronary artery disease. Due to substantial technological advancement, refinement in clinical imaging protocols, the fast-growing feasibility of CT, and low patient radiation exposures, CCTA rivals conventional catheter angiography in the ability to illustrate coronary artery anatomy [8]. Moreover, due to its high sensitivity and specificity, CCTA allows accurate diagnosis of CAD when compared, for instance, to functional stress testing [9,10,11]. Given the high diagnostic accuracy of coronary CTA [12,13], it is vital to determine how to best improve the diagnostic yield of CCTA without unnecessary overutilization. The indications for CCTA must be set so that risks and benefits for a single patient are balanced. Inappropriate use of CCTA may be potentially harmful to patients and lead to unnecessary costs to the health care system [14,15,16]. Therefore, the purpose of our study was to retrospectively assess the appropriateness of the use of CCTA as defined in existing board-certified recommendations in correlation with invasive coronary angiography (ICA). 

## 2. Methods 

### 2.1. Study Population

**Figure 1:**
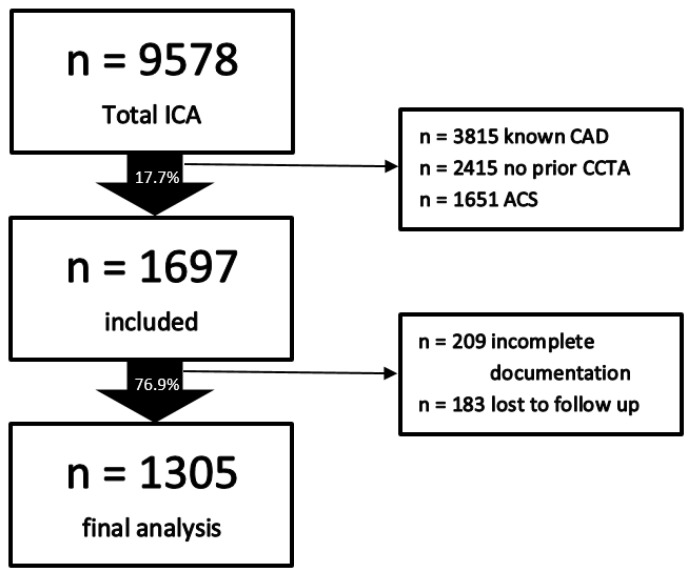
Study design. Abbreviations: ACS—acute coronary syndrome, CAD—coronary artery disease, CCTA—coronary computed tomography angiography, ICA—invasive coronary angiography.

A total of 9578 patients were retrospectively screened over 4 years; 7881 were excluded due to known CAD, lack of previous CCTA, and ACS. Of the remaining 1697 patients, 392 were excluded due to incomplete documentation and lack of follow-up. Thus, data from 1305 patients referred to ICA after pathologic CCTA were included in our final analysis. We defined a pathologic CCTA as a stenosis of >50% in one or more epicardial vessels. The indication of CCTA was made by board-certified cardiologists or specialists in internal medicine from primary, secondary, or tertiary referral centers. The study was approved by our local institutional review board; patients’ informed consent forms were waived. A flowchart illustrating patient selection is shown in Figure 1.

### 2.2. Inclusion/Exclusion Criteria

Inclusion criteria: age > 18 years, CCTA prior to invasive angiography, complete documentation. Exclusion criteria: known CAD, previous invasive coronary angiography (ICA), percutaneous coronary intervention (PCI), coronary artery bypass graft (CABG), acute myocardial infarction, unstable angina, renal insufficiency (GFR < 30 mL/m²/min), hyperthyroidism, iodine allergy, pregnancy. 

### 2.3. Coronary Computed Tomography Angiography and Definition of Obstructive CAD 

CCTA was performed using different scanner types with a minimum of 64 slices. The assessment was made by one experienced observer (10 years of training, equivalent to ACCF/AHA level 3 accreditation) or a resident in cardiac imaging training with subsequent discussion by an experienced level 3 observer. Obstructive CAD was defined as a stenosis > 50% in one or more epicardial vessels. 

### 2.4. Clinical Definitions/Procedure

All previous imaging exams and reports were acquired, and the indication for CCTA was reviewed and retrospectively classified as appropriate (A), uncertain (U), or inappropriate (I) following consensus papers [10,15].

Cardiovascular risk factors (diabetes, arterial hypertension, family history of premature cardiovascular death, dyslipidemia, and smoking) were gathered for each patient. Symptoms (according to the Canadian Cardiovascular Society (CCS) scale and New York Heart Association (NYHA) score) at the time of ICA referral were categorized, and the patients’ respective pre-test probability was calculated according to the modified Diamond and Forrester plot. Clinical follow-up was performed using patient chart reviews and included ICA results and revascularization rate.

### 2.5. Statistical Analysis

To perform statistical analyses, SSPSTM software (version 17.0, SPSS Inc, Chicago, IL, USA) was used. Quantitative variables are expressed as mean ± SD. In case of normal distribution, further comparisons were made using *t*-test or one-way ANOVA. Categorical variables are given as absolute values and percentages and were compared with Chi-squared and Fisher’s exact test as appropriate. Multivariate linear regression analysis was performed to adapt for significant differences in known CAD risk factors. A statistically significant difference was defined as a *p* value < 0.05. 

## 3. Results

The final analysis included 1305 patients (mean age years 63.9; 31.9% female) referred to ICA due to pathological CCTA. Of those, the CCTA indications of 496 (38.0%) were appropriate, 43 (3.3%) were uncertain, and 766 (56.9%) were inappropriate (Table 1 displays patient characteristics).

Considering symptoms, age, and sex that defined the respective pre-test probability for epicardial coronary artery disease, we found that 48.3% (*n* = 370) of the referrals defined as inappropriate were asymptomatic or presented with atypical chest pain classified as CCS 0. 

A total of 396 patients (51.7%) presenting with more typical angina, dyspnea, or mixed symptoms (combination of CCS or/and NYHA classifications), adjusted for age and sex and with the addition of existing risk factors, had a higher pre-test probability and should have been referred to functional testing or directly to invasive diagnostic angiography. 

The overall mean age was 63.9 ± 9.6, with a significantly lower mean (52.6 ± 5.8) in uncertain referrals compared to appropriate (62.2 ± 9.3) and inappropriate (65.6, *p* < 0.001). Overall cardiovascular risk profiles were intermediate–high in all appropriateness groups; there was a significant difference between inappropriate and appropriate referrals in female patients (47.2% appropriate, 100% uncertain, and 18.1% inappropriate, *p* < 0.001).

**Figure 2:**
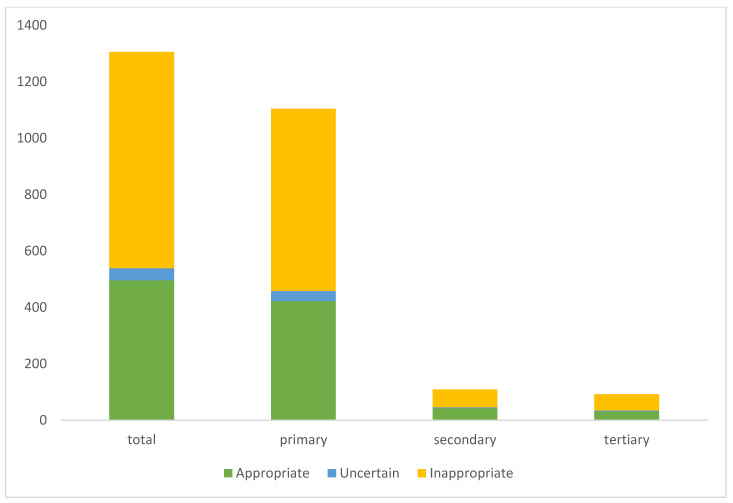
Absolute numbers of referrals for each appropriate use criteria in relation to referral source.

There were no significant differences between the groups in each group considering the referring sources (tertiary, secondary, or primary care facilities): of 766 inappropriate referrals, 57 (7.4%) were admitted by a tertiary center, 63 (8.2%) by a secondary, and 646 (84.3%) by a primary care physician. On the other hand, most appropriate referrals also came from primary referring sources. The absolute numbers of patients attributed to the three categories of appropriateness and the respective referral sources are displayed in Figure 2.

The CAD pre-test probability rates were calculated following adapted ESC guidelines [5] defining appropriate coronary CT imaging referrals in patients with an intermediate probability and are displayed in Table 1. 

**Figure 3:**
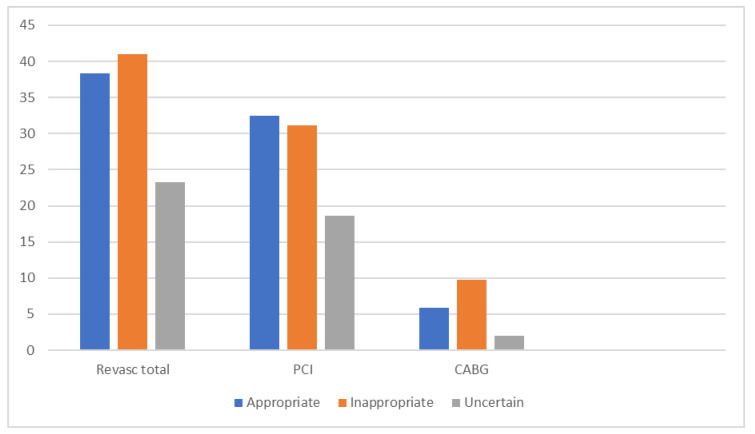
Total revascularization split in percutaneous coronary intervention (PCI)/coronary artery bypass graft (CABG). Abbreviations: CABG—coronary artery bypass graft, PCI—percutaneous coronary intervention. The total revascularization rate was 39.2%, of which were 79.5% PCI and 20.5% CABG, without significant differences between the groups (*p* value = 0.071).

The total coronary revascularization rate was 39.2%, resulting in 79.5% PCI and 20.5% CABG), without differences between the groups (*p* = 0.071) (Table 2 and Figure 3).

Interestingly, coronary revascularization rates did not differ significantly between the appropriate and the inappropriate groups (38.3% vs. 41%, *p* = 0.068). The rates of percutaneous coronary interventions were similar; the rate of CABG was higher in the inappropriate group.

## 4. Discussion

Suspected CAD is a leading global health problem and one of the most common diagnostic problems clinicians encounter. Results of previous data indicate an existing suboptimal use of CCTA in various institutions based on rates of inappropriate use between 5% and 25% [17]. Our data show an even higher amount of inappropriate CCTA indications. The best CCTA assessment should involve ruling out ischemic coronary disease. We saw a major portion of inappropriate diagnoses due to a too-low (but mostly to a too-high) CAD pre-test probability in regard to existing guidelines. For example, 2013 recommendations did not advise the use of coronary CTA screening in patients outside the 15–50% pre-test probability window, which represents an intermediate risk cohort [5]. 

Surprisingly, the rate of significant coronary stenoses and subsequent revascularization was comparable to appropriately referred patients.

Smaller single-center studies [17,18] showed a resemblance to this observation. Yang and colleagues found an inappropriate rate of 8.1% in 402 patients with a comparable proportion of subsequent revascularization. However, the rate of invasive angiography was higher compared to the appropriately referred cohort. The evaluation from a single center in Bahrain yielded an inappropriate rate of 4.7% in 234 CT studies but did not report on downstream testing [18].

A similar portion of patients was inappropriately referred for CCTA with a too-high pre-test probability. In this group, CCTA does not add any further diagnostic information according to existing guidelines. Additional ischemia testing or direct referral to invasive angiography would have been a better choice for these patients.

We furthermore see a small number of patients with uncertain CCTA indications (3.3%). The previously cited study from Bahrain found a higher number of uncertain CCTA indications (20.1%) but applied a different rating system [18]. The major part of their uncertain referrals were asymptomatic patients with a high risk of CAD, which would be reclassified as inappropriate according to the current ESC guidelines or the appropriate use criteria we used for this analysis. 

Nevertheless, this highlights the high uncertainty in clinical decision making in an insufficiently attributed patient cohort that requires the physician’s judgment to determine the pathway for an individual clinical scenario [14].

In accordance with the guidelines, asymptomatic patients not requiring CCTA could be further risk-stratified with a simpler approach using coronary calcium scanning added to various standard risk scores [19,20,21,22]. Yang et al. developed a scoring system based on various clinical variables to identify patients at high and low risk of CAD. They found an improved distinction between patients who benefit more from primary preventive drug therapy or from further invasive diagnostic/therapy. Compared to the Diamond–Forrester model, their score performed significantly better without overlapping confidence intervals [23].

The rate of patients with significant CAD in ICA after inappropriate use of CCTA was high. Revascularization in these patients was performed at the discretion of the operator or the heart team’s decision. This may be explained by the definition of appropriate and inappropriate CCTA indications. The consideration of the high number of inappropriate CT referrals only included patients with no, atypical, or typical symptoms, which may explain the unexpected ICA-triggered numbers of revascularization procedures. We see this underlined by a more precise clinical evaluation of chest pain symptoms with newly defined angina classification and the introduction of dyspnea as an additional feature in the newer chronic coronary syndrome ESC guidelines [6].

Of our patients, 105 had left main or/and severe three-vessel disease requiring subsequent CABG. Previous studies in asymptomatic patients, mainly diabetes subsets, revealed high rates of obstructive CAD in CCTA [24,25]. This highlights the necessity to reconsider pre-test probability models based on symptom evaluation, like that described above, and fostering further referrals to ischemia testing.

In addition, our patient cohort, preselected due to the retrospective approach, shows high cardiovascular risk profiles. Therefore, the proportion of patients referred to CCTA due to a too-low initial probability of CAD (classified as inappropriate) and undergoing subsequent revascularization was as high as 36%. The distribution of these risk factors was similar in the appropriate and inappropriate groups, causing a preselection bias.

Another issue is that we depend on a large diversity of referring sources and cannot judge how accurate individuals’ knowledge of existing appropriate CT guidelines is. Moreover, the timing of easily available CT investigation before invasive angiography was certainly co-influenced by longer schedule delays of other imaging or functional ischemia testing modalities. 

## 5. Limitations

This study was performed retrospectively with an inherent bias of only including patients after ICA and correlating their results with previous CCTA findings. In a second step, the appropriateness of the CCTA referrals was investigated. We therefore have a predefined patient population but wanted to draw a real-world clinical scenario. 

A second limitation is that the study design is single-centered, but the large number of patients and the variety of CCTA referring physicians were an elementary step of the investigation. A large number of CCTA patients with nonpathologic reports not referred to ICA was excluded and could have served as comparing group; nevertheless, this group would only be comparable in terms of the need for revascularization. This population, of course, was guided to primary prophylactic measures and/or medical treatment depending on the CCTA reports.

## 6. Conclusion

In our single-center evaluation, CCTA use diverged from the current appropriate use criteria. We observed a large number of patients with obstructive CAD and subsequent revascularization, regardless of the initial appropriateness recommendation. CCTA, therefore, may have an incremental prognostic and clinical value in patients beyond the intermediate pre-test probability classification and should be considered a valuable diagnostic tool. It should be pointed out, however, that a more precise exploration and definition of clinical chest pain symptoms may avoid inappropriate CCTA referrals, especially in higher pre-test probability populations. 

## Figures and Tables

**Table 1:** Clinical characteristics and CAD pre-test probability calculations.

	Total (*n* = 1305)	Uncertain (*n* = 43; 3.3%)	Appropriate (*n* = 496; 38%)	Inappropriate (*n* = 766; 56.9%)	*p* Value
Mean age, years	63.9 ± 9.6	52.6 ± 5.2	62.2 ± 9.3	65.6 ± 9.4	<0.001
Female	416 (31.9)	43 (100.0)	234 (47.2)	139 (18.1)	<0.001
BMI	27.2 ± 4.4	27.5 ± 5.8	26.9 ± 4.7	27.3 ± 4.0	0.75
Dyslipidemia LDL	874 (67.0) 121.1 ± 33.2	23 (53.5) 118.3 ± 49.2	346 (69.8) 135.2 ± 38.0	505 (65.9) 117.7 ± 34.2	0.05
Hypertension Systolic BP	987 (75.6) 140.3 ± 16.7	28 (65.1) 136.9 ± 19.1	370 (74.6) 140.1 ± 17.1	589 (76.9) 141.3 ± 16.4	0.10
Smoker	543 (41.6)	21 (48.8)	218 (44.0)	304 (39.7)	0.33
Diabetes mellitus HbA1c	180 (13.8) 5.6 ± 0.6	7 (16.3) 5.8 ± 1.1	61 (12.3) 5.7 ± 0.8	112 (14.6) 5.5 ± 0.7	0.77
Positive FH	429 (32.9)	21 (48.8)	171 (34.5)	237 (30.9)	0.099
Referring sources					0.89
Tertiary	92 (7.0)	4 (9.3)	31 (6.3)	57 (7.4)	
Secondary	109 (8.4)	3 (7.0)	43 (8.7)	63 (8.2)	
Primary	1104 (84.6)	36 (83.7)	422 (85.1)	646 (84.3)	
CCS					<0.001
Angina classification					
0	533 (40.8)	8 (18.6)	113 (22.8)	412 (53.8)	
I	301 (23.1)	28 (65.1)	211 (42.5)	62 (8.1)	
II	240 (18.4)	7 (16.3)	148 (29.8)	85 (11.1)	
III	117 (8.9)	0	19 (3.8)	98 (12.8)	
IV	114 (8.7)	0	5 (1.0)	109 (14.2)	
NYHA					<0.001
0	25 (1.9)	1 (2.3)	11 (2.2)	13 (1.7)	
I	761 (58.3)	18 (41.9)	190 (38.3)	553 (72.2)	
II	414 (31.7)	21 (48.8)	256 (51.6)	137 (17.9)	
III	92 (7.0)	3 (7.0)	338 (6.7)	56 (7.3)	
IV	13 (1.0)	0	6 (1.2)	7 (0.9)	
Pre-test probability					<0.001
Low	413 (31.6)	43 (100.0)	0	370 (48.3)	
Intermediate	496 (38.0)	0	496 (100.0)	0	
High	396 (30.3)	0	0	396 (51.7)	

Abbreviations: BMI—body mass index, BP—blood pressure, CCS—Canadian Cardiovascular Society scale, FH—family history, LDL—low-density lipoprotein, NYHA—New York Heart Association score. Presented data are mean values and standard deviations for variables approximately normal.

**Table 2:** Coronary revascularization rates in the different appropriateness CCTA referral groups.

Total *n* = 1305	Uncertain *n* = 43	Appropriate *n* = 496	Inappropriate *n* = 766	*p* Value
Revascularization *n* = 512 (39.2%)	10 (23.3%)	190 (38.3%)	311 (40.6%)	0.068
PCI *n* = 407	8 (18.6%)	161 (32.5%)	238 (31.1%)	0.17
CABG *n* = 105	2 (4.7%)	29 (5.9%)	74 (9.7%)	0.037

Abbreviations: CABG—coronary artery bypass graft, PCI—percutaneous coronary intervention.

## Data Availability

Not applicable.

## Abbreviations

A	Appropriate
ACS	Acute coronary syndrome
CABG	Coronary Artery Bypass Graft
CCSC	Canadian Cardiovascular Society Classification
CCTA	Computed Coronary Tomographic Angiography
CTA	Computed Tomographic Angiography
CAD	Coronary Artery Disease
GFR	Glomerular Filtration Rate
I	Inappropriate
ICA	Invasive Coronary Angiography
NYHA	New York Heart Association
PCI	Percutaneous Coronary Intervention
U	Uncertain
